# DISPARITIES OF DENTAL CARE UTILIZATION AMONG WHITE, BLACK, HISPANIC, AND ASIAN FROM 2002 TO 2016

**DOI:** 10.1093/geroni/igz038.2258

**Published:** 2019-11-08

**Authors:** Yanyan Wu, Wei Zhang, Bei Wu

**Affiliations:** 1 University of Hawaii at Mānoa, Honolulu, Hawaii, United States; 2 New York University, New York, New York, United States

## Abstract

Utilizing eight waves (2002-2016) of data from the Behavioral Risk Factor Surveillance System (BRFSS), this study examines the disparities and trend of dental care utilization among dentate adults aged 50 years or older in the U.S. Findings reveal that there was a significant increase of dental care utilization among Asians and Blacks in older adults. The rates increased from 73% (95%CI: 69%-78%) to 81% (95%CI: 79%-84%) and 54% (95%CI: 52%-56%) to 60% (95%CI: 69%-62%) for Asians and Blacks respectively in the 75-79 age-group. Early in 2002, there was a linear decreasing pattern of dental care utilization with age. However, the 65-69 age-group became a turning point in 2018 with a lower rate of dental care utilization before 65-69 years of age and higher rate in the older age groups. Overall, Hispanics and Blacks had much lower rates of dental care utilization than that of Whites and Asians.

